# Predicting nosocomial infections in critically Ill children: a comprehensive systematic review of risk assessment models

**DOI:** 10.3389/fped.2025.1636580

**Published:** 2025-09-10

**Authors:** Ling-Ying Wang, Mei Feng, Yu-Lan Luo, Chun-Xia Wang, Heng Wang, Li Li, Yuan Zhang, Xiu-Ling Huang, Min-Jie Huang, Yong-Ming Tian

**Affiliations:** ^1^ Department of Critical Care Medicine, West China Hospital, Sichuan University/West China School of Nursing, Sichuan University, Chengdu, China; ^2^ Nursing Department, West China Hospital, Sichuan University/West China School of Nursing, Sichuan University, Chengdu, China; ^3^ Department of Anesthesiology, West China Hospital, Sichuan University, Chengdu, China

**Keywords:** prediction, nosocomial infections, intensive care unit, child, model

## Abstract

**Background:**

Nosocomial infections (NIs) pose a substantial global health challenge, affecting an estimated 7%–10% of hospitalized patients worldwide. Neonatal intensive care units (NICUs) are particularly vulnerable, with NIs representing a leading cause of infant morbidity and mortality. Similarly, pediatric intensive care units (PICUs) report that 28% of admitted children acquire NIs during hospitalization. Although prediction models offer a promising approach to identifying high-risk individuals, a systematic evaluation of existing models for ICU-ill children remains lacking.

**Aim:**

This review systematically synthesizes and critically evaluates published prediction models for assessing NI risk in ill children in the ICU.

**Methods:**

We conducted a comprehensive search of PubMed, Embase, Web of Science, CNKI, VIP, and Wanfang from inception through December 31, 2024. Study quality, risk of bias, and applicability were assessed using the PROBAST tool. Model performance metrics were extracted and summarized.

**Results:**

Three studies involving 1,632 participants were included. Frequency analysis identified antibiotic use, birth weight, and indwelling catheters as the most consistently incorporated predictors. All models employed traditional logistic regression, with two undergoing external validation. However, critical limitations were observed across studies: inadequate sample sizes, omission of key methodological details, insufficient model specification, and a universally high risk of bias per PROBAST assessment.

**Conclusion:**

Current NI prediction models for ill children in the ICU exhibit significant methodological shortcomings, limiting their clinical applicability. No existing model demonstrates sufficient rigor for routine implementation. High-performance predictive models can assist clinical nursing staff in the early identification of high-risk populations for NIs, enabling proactive interventions to reduce infection rates. Future research should prioritize (1) methodological robustness in model development, (2) external validation in diverse settings, and (3) exploration of advanced modeling techniques to optimize predictor selection. We strongly advocate adherence to TRIPOD guidelines to enhance predictive models' transparency, reproducibility, and clinical utility in this vulnerable population.

**Systematic Review Registration:**

PROSPERO CRD420251019763.

## 1 Introduction

Over seven decades of development, critical care medicine has undergone transformative, industrial-scale advancements in managing life-threatening conditions. However, mirroring the unintended consequences of industrialization, these breakthroughs- particularly for vulnerable populations (e.g., elderly, immunocompromised, and chronically ill patients) - have inadvertently increased the prevalence and complexity of nosocomial infections (NIs) in modern intensive care units (ICUs) (1). The US Center for Disease Control and Prevention identifies that nearly 1.7 million hospitalized patients annually acquire NIs while being treated for other health issues and that more than 98,000 patients (one in 17) die due to these (2). The burden of NIs demonstrates marked geographical inequalities. Lower- and middle-income countries (LMICs) report an average NI prevalence of 17%, with surgical site infections constituting the predominant type, followed by urinary tract infections, bloodstream infections, then respiratory tract infections (3). In striking contrast, high-income countries (HICs) document NI rates approaching 30% among ICU patients - a phenomenon attributable primarily to three key factors: (1) higher prevalence of patient comorbidities, (2) greater use of immunosuppressive therapies, and (3) more frequent employment of invasive medical devices (3, 4).

NIs in neonatal intensive care units (NICUs) represent a particularly alarming global health concern, significantly contributing to infant morbidity and mortality worldwide (5). Premature neonates are confronted with a host of compounded vulnerabilities: prolonged hospitalizations, immature immune systems, fragile skin integrity, and frequent exposure to life-saving yet infection-prone medical interventions (5–7). While the United States witnessed significant reductions in central line-associated bloodstream infections (CLABSIs) between 2007 and 2012, recent progress in this area has unfortunately stagnated (8, 9). In contrast, in low- and middle-income countries (LMICs), neonatal nosocomial infections (NIs) impose a disproportionately higher burden, mainly due to resource constraints and care disparities (10). Globally, in pediatric intensive care units (PICUs), 28% of children develop nosocomial infections during their stay (11). In such critical care settings, the timely identification of high-risk child patients and the implementation of preventive interventions are of paramount importance. Complications such as prolonged length of stay in ICU, prolonged length of stay in hospital, excess hospitalization costs, and predicted mortality are highly associated with NIs (12, 13), and place a substantial economic burden on healthcare systems (14). Fortunately, modifiable care processes present actionable targets for reducing infection prevalence, offering hope for meaningful improvements in patient outcomes and healthcare efficiency.

In clinical settings, the current identification of NIs primarily relies on specimen culture-based diagnostics, which typically require several days to produce results. This delay often necessitates initiating empirical broad-spectrum antibiotic therapy, exposing non-infected patients to unnecessary treatments and exacerbating the selective pressure for antibiotic-resistant pathogens (15). Moreover, traditional NI risk stratification remains inherently subjective, as it depends on clinician experience and qualitative interpretations of patient data. This subjectivity leads to inconsistent decision-making and limited reproducibility across care providers (16). Such variability compromises the reliability of correlating clinical risk factors with prognostic outcomes, further underscoring the critical role of practitioner expertise in contextualizing patient-specific scenarios. Clinical prediction models (CPMs) offer a promising alternative by providing objective, algorithm-driven frameworks that integrate multidimensional risk factors into statistical models to estimate disease probability or event risk in defined populations (17–19).

By leveraging historical patient data, data-driven decision support systems can effectively mitigate cognitive biases in clinical judgment, delivering standardized prognostic insights that enhance care precision (20). However, while numerous CPMs have been developed for NI risk prediction in adult ICUs, pediatric applications in neonatal and pediatric critical care populations remain significantly underdeveloped. Existing pediatric models often lack systematic evaluation, comparative benchmarking, or consensus-based validation for clinical implementation. Additionally, the methodological rigor across studies varies widely, necessitating rigorous appraisal to establish evidence-based recommendations for practice.

To address these gaps, this study conducts a comprehensive performance evaluation of all published, validated NI risk prediction models specific to ill children in ICUs. Through structured comparison and quality assessment, we aim to identify optimal predictive tools for clinical translation and formulate prioritized research directions to advance NI risk stratification in critically ill children.

## 2 Methods

### 2.1 Design

This study adhered to the Critical Appraisal and Data Extraction for Systematic Reviews of Prediction Modelling Studies (CHARMS) checklist to systematically evaluate predictive modeling studies (21). The review was planned and reported by the PRISMA 2020 guidelines (22). The protocol was registered on the International Prospective Register of Systematic Reviews (PROSPERO, ID: CRD420251019763).

### 2.2 Search strategy

We conducted a comprehensive systematic search across six major databases: PubMed, Web of Science, Embase, China National Knowledge Infrastructure (CNKI), Chinese Technical Periodicals (VIP), and Wanfang databases, covering their inception to December 2024. The search strategy incorporated relevant medical subject headings (MeSH) and free-text terms related to ICU, infection control, and predictive modeling. A detailed description of the complete search strategy is provided in the Supplementary Table S1. Additionally, the reference lists of all included studies and relevant reviews were reviewed to identify any additional references.

### 2.3 Study eligibility criteria

We established the inclusion and exclusion criteria before the study.

#### 2.3.1 Inclusion criteria

1. Study population: Children aged under 18 years.
2. Study content: Studies on risk prediction models for NIs in ICU child patients (including neonatal and pediatric populations).
3. Study type: Case-control studies and cohort studies.

#### 2.3.2 Exclusion Criteria

1. Studies focusing on adult populations (age ≥ 18 years).
2. Non-longitudinal study designs.
3. Models with fewer than two predictors.
4. Duplicate publications.
5. Unofficial publications, such as conference abstracts and academic papers.
6. Studies not available in Chinese or English.

### 2.4 Study selection

We imported the search results into EndNote X9 software for data management. After removing duplicates, the titles and abstracts of the retrieved studies were screened. Full texts of potentially eligible studies were then obtained and evaluated. Two authors independently assessed all studies, with any disagreements resolved through discussion with additional review authors. Multiple publications from the same model were compiled, with the most comprehensive report designated as the primary reference.

### 2.5 Data extraction

Data extraction was independently conducted by two authors using Microsoft Excel, with discrepancies resolved through consultation with other review authors. Standardized data extraction forms were designed based on the CHARMS checklist (21). The critical information extracted followed the PICOTS principles, including the number of subjects included, data source, predictors (e.g., age, albumin infusion), model status (e.g., performance, modeling status, and model presentation), and outcome metrics. We collected information such as author names, year of publication, study type, and statistical details (e.g., treatment of missing data, selection of predictors, and treatment of continuous variables).

To analyze the predictive ability of each model, the following metrics were used to evaluate the clinical applicability:

1. Discrimination: The model's ability to distinguish between individuals with and without the outcome of interest, often measured by the Consistency Statistics (C-index) and the Area Under the Curve (AUC). The closer the AUC is to 1, the better the diagnostic effectiveness of the model (23);
2. Calibration: The accuracy of probability predictions, measured by the Hosmer-Lemeshow test and calibration curves (24);
3. Clinical Validity Evaluation Metrics: Decision Curve Analysis (DCA) was used to assess the clinical utility of predictive models, aligning with practical clinical decision-making processes (25). In addition to these metrics, the confusion matrix, accuracy, sensitivity, specificity, F1-score, and Brier score were also evaluated (26).

### 2.6 Quality assessment

We applied the Risk of Bias Assessment Tool (PROBAST) (27) to assess the risk of bias (ROB) and the applicability of the prediction models. PROBAST comprises four domains: participants, predictors, outcomes, and analysis. Each question can be answered as “yes,” “probably yes,” “probably no,” “no,” or “no information.” A domain was considered high risk if any question was answered “no” or “probably no.” Conversely, a domain was defined as low risk if all questions were answered “yes” or “probably yes.” The overall ROB was deemed low when each domain consistently exhibited a low ROB. If one or several domains exhibited an uncertain ROB while the remaining domains were low risk, the ROB was categorized as unclear. The applicability evaluation followed a similar approach, but only the first three domains were used to assess the applicability of the predictive model. Two authors independently evaluated the risk of bias in the included models, with any discrepancies resolved through consultation with other review authors.

### 2.7 Data synthesis and analysis

Given the significant heterogeneity in study design, populations, and outcomes across the studies, we decided against pooling data for meta-analysis. This decision ensured that the unique findings of each study, reflecting diverse study designs and population characteristics, were preserved.

## 3 Results

### 3.1 Study selection and characteristics

**Figure 1:**
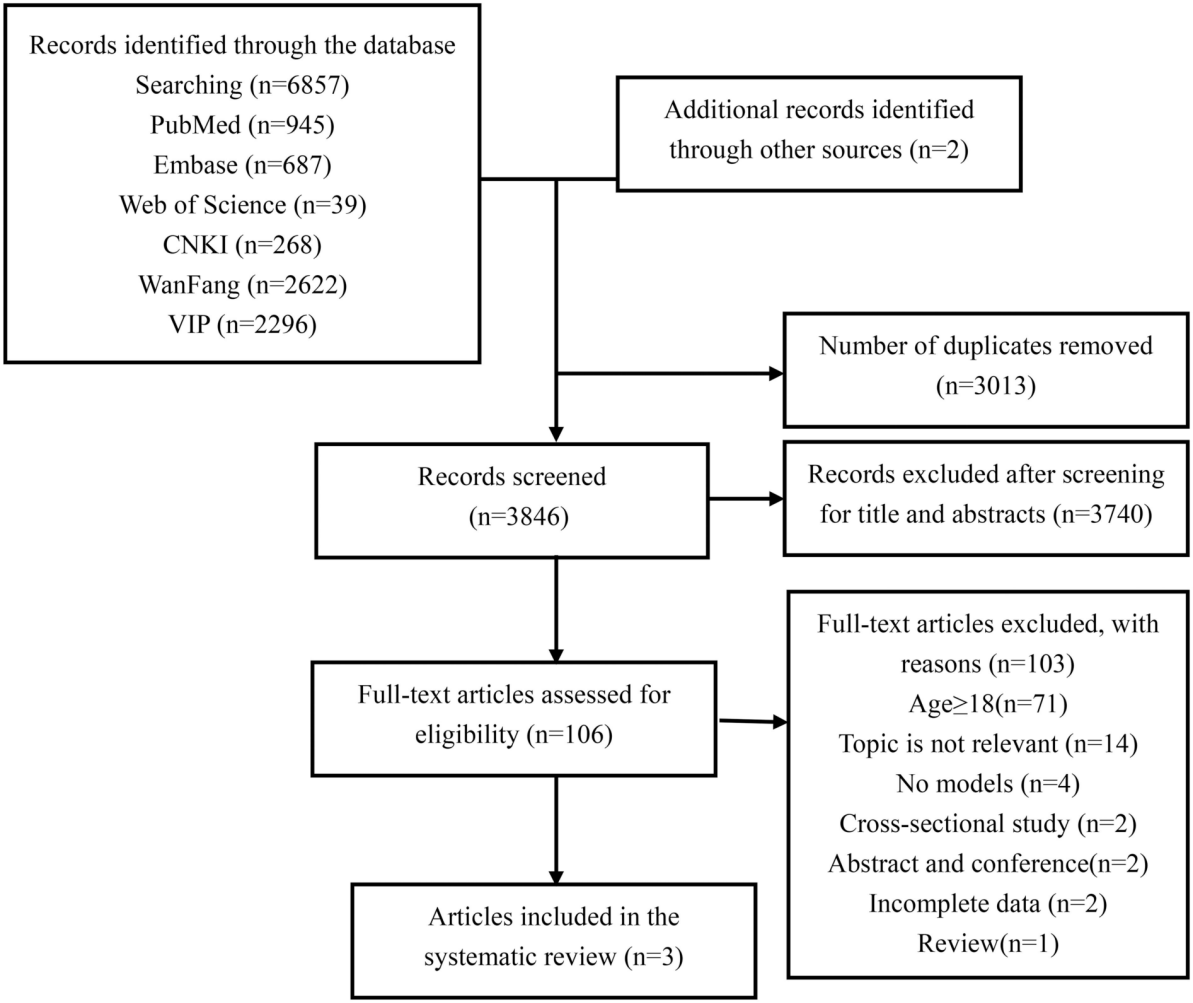
Flowchart of the selection of studies for inclusion in the systematic review.

The PRISMA flowchart illustrates our process for searching and selecting literature (Figure 1). A total of 6,857 articles were identified in the initial search. After removing duplicates, 3,846 articles remained for title and abstract screening. Full-text reviews were conducted on 106 articles, of which only three were ultimately included in this review (28–30).

### 3.2 Characteristics of the included studies

The characteristics of the included studies are summarized in Table 1. The three studies were published between 2016 and 2023 and conducted across Asia, specifically in China and India.

**Table 1:** Characteristics of the included studies and model development.

Author/year	Country	Design/sample size	Setting/modeling group (event rate)	Outcome/measurement	Variables selection methods	Modelling method	No./predictors in the final model (*β* coefficient)	Validation	Presentation
Zhou/2023 (28)	China	Retrospective cohort/549	NICU/infect group:89, Non-infect group: 346 (9.71%)	MDRO/MDROs mainly refer to bacteria resistant to three or more antibiotics clinically, including VRE, CRE, MRSA, CRPA, CRAB, and ESBLs -producing Enterobacteriaceae (e.g., Escherichia coli and Klebsiella pneumoniae), etc. According to the < Diagnostic criteria for nosocomial infections (trial)> issued by the Ministry of Health, China, nosocomial infections refer to infections acquired in the hospital by hospitalized patients, including infections occurred during hospitalization or acquired in the hospital and occurred after discharge. However, nosocomial infections do not include infections that occurred before admission or existed at admission	univariate analysis	Logistic regression	4/A: use of antibiotics >7 days (1.498), B: maternal age ≥35 years (1.435), C: low birth weight (1.089), D: MDRO colonization (0.790)	Internal validation: Bootstrap AUC: 0.773 (95% CI: 0.718 ∼ 0.828) Calibration: H-L: *p* = 0.61, calibration curves DCA Temporal external validation: AUC: 0.788 (95% CI: 0.677 ∼ 0.899) Calibration: H-L:*P* = 0.49, calibration curves DCA sensitivity: 64.71%, specificity: 79.38%, the Jordan index: 0.44, prediction accuracy: 77.19%	Nomogram
Miao/2023 (29)	China	Retrospective cohort/672	NICU/infect group:34 Non-infect group: 413 (7.6%)	Umbilical vein catheterization bloodstream infection/According to the Guidelines for Prevention and Control of Complications Associated with Umbilical Venous Catheterization in Neonates: A diagnosis of umbilical venous catheter-related bloodstream infection should be made when: 1. A neonate develops significant signs of infection (such as fever, chills, or hypotension) within 48 h after umbilical venous catheter placement or removal; 2. No other identifiable source of infection is found except the catheter; 3. Paired blood cultures (from the catheter and peripheral blood) yield the same pathogen; and 4. The catheter blood culture turns positive more than 2 h earlier than the peripheral blood culture. For microbiological confirmation: 1. Bacterial isolation and culture were performed in strict accordance with the National Clinical Laboratory Procedures; 2. Bacterial identification was conducted using the VITEK 2 Compact automated microbial analysis system (bioMérieux, France) with matched identification and antimicrobial susceptibility testing cards.	Univariate analysis	Logistic regression	6/A: serum albumin <35 g/L (1.854), B: puncture attempts >2 times (1.749), C: birth weight <1,500 g (1.607), D: history of PICC treatment (1.382), E: The total indwelling time >7 days (0.989), F: history of mechanical ventilation (0.950)	Internal validation: AUC: 0.866 (95% CI: 0.784–0.947) Calibration: H-L:P = 0.323, calibration curves Sensitivity: 0.853, specificity: 0.789, the Jordan index: 0.642 Temporal external validation: AUC 0.837 (95% CI: 0.744–0.930) Sensitivity: 0.700, specificity: 0.849), the Jordan index: 0.549	Nomogram
L.G. Saptharishi /2016 (30)	India	Prospective study/411	PICU/ 151 episodes of HAIs (in 95 children; 23.1%), with an absolute event rate of 4.5 per 100 patient-days.	HAI/HAIs were diagnosed based on the (CDC surveillance definitions (Supplementary Appendix 1) in the children after 48 h of PICU admission and within 72 h of transfer from PICU. For the derivation of the score, only those with positive microbiological results (culture-positive cases) from relevant body fluids were included.	univariate analysis	Logistic regression	7/ A: need for intubation (1.70), B: presence of indwelling catheters (1.67), C: albumin infusion (1.35), D: immunomodulator (1.30), E: Age (<5 years) (0.90), F: prior antibiotic use (≥4) (0.58), G: Pediatric Risk of Mortality III (24 h) score (0.06)	**Internal Validation:** Bootstrap ROC: 0.87. Calibration: H-L: *P* = 0.75 Sensitivity: 79.1% Specificity: 79.1% Positive predictive value: 61.6% Negative predictive value: 89.9%, accuracy: 79.3%	Pediatric Risk of Nosocomial Sepsis score (PRiNS Score)

MDRO, multi-drug resistant organism; VRE, vancomycin-resistant Enterococcus; CRE, carbapenems-resistant Enterobacteriacea; MRSA, methicillin-resistant Staphylococcus aureus; CRPA, carbapenem-resistant Pseudomonas aeruginosa; CRAB, carbapenem-resistant Acinetobacter baumannii; ESBLs, extended-spectrum beta-lactamases; PICC, Peripherally Inserted Central Catheter; HAI, health care–associated infections; CDC, Center for Disease Control and Prevention; N/A, not applicable; H-L, Hosmer-Lemeshow.

#### 3.2.1 Study design and population

The modeling research designs were predominantly retrospective (28, 29), with one study employing a prospective design (30). The sample sizes of the two retrospective studies were 549 and 672, respectively, while the prospective study included 411 participants. One study focused on patients in the PICU aged 1 month to 12 years (30), while the other two involved NICU patients (28, 29).

#### 3.2.2 Outcomes

The outcome measures in the three included studies were not identical. Zhou et al. (28) used multidrug-resistant organisms (MDROs) as the outcome measure for modeling. Miao et al. (29) focused on umbilical vein catheterization bloodstream infections, while Saptharishi L. G. et al. (30) used healthcare-associated infections (HAIs) as the outcome measure for modeling.

#### 3.2.3 Predictors selection

All three studies used univariate analysis to select the most relevant factors for their models. A total of 17 final variables were identified across the studies, as shown in Table 1. Among the 17 predictors, three variables were most consistently incorporated across studies: antibiotic use (included in 2 studies): “use of antibiotics >7 days” (28) and “prior antibiotic use (≥4)” (30) were identified in both NICU and PICU models. Birth weight (included in 2 studies): “low birth weight” and “birth weight <1,500 g” (28, 29) were exclusive to NICU models. Indwelling catheters (included in 2 studies): “total indwelling time >7 days” (29) and “presence of indwelling catheters” (30) were featured in both NICU and PICU models.

Other predictors were included in only one study, including maternal age ≥35 years, MDRO colonization, serum albumin <35 g/L, puncture attempts >2 times, history of mechanical ventilation, history of peripherally inserted central catheter (PICC) treatment, age (<5 years), pediatric risk of mortality III (PRISM III) score (within 24 h), need for intubation, albumin infusion, and immunomodulator use, reflecting variability in study populations (NICU vs. PICU) and outcomes (MDROs vs. catheter-related infections vs. HAIs).

#### 3.2.4 Missing data handling

In the study by Zhou et al., a total of 459 cases of nosocomial infections were detected in the modeling cohort. After excluding 24 cases (5.2%) with incomplete data, 435 neonates were finally included to construct the prediction model; the cases in the validation cohort had complete data, with no exclusions (28). In the study by Miao et al., the completeness of clinical data was set as an inclusion criterion. In the modeling group, 17 cases (3.7%) were excluded due to missing clinical and laboratory data, and 6 cases (2.6%) were excluded in the validation group (29). In the study by Saptharishi et al., among the 412 initially screened eligible subjects who signed the informed consent form, 1 case (0.2%) with incomplete data was excluded, and finally 411 cases were included in the model construction (30).

#### 3.2.5 Modeling methods and validation

All three studies utilized multivariate logistic regression for modeling. Two studies conducted internal and external validation after model establishment (28, 29), while the other research combined model construction with internal validation using the Bootstrap method (30). Discrimination and calibration were reported for all three models, and only one model used DCA to assess clinical validity (28). The discrimination AUC values of the internally validated models ranged from 0.773 to 0.866.

#### 3.2.6 Model presentation and reporting standards

Two of the models were presented as nomograms (28, 29), while the presentation format of the third model was scoring table (30). The Transparent Reporting of a Multivariable Prediction Model for Individual Prognosis or Diagnosis (TRIPOD) guidelines were published in 2015 to standardize the reporting of prediction model research, ensuring transparency and reproducibility (19, 31). Although all three included studies were published after 2015, none explicitly stated that they followed the TRIPOD statement. The completion status of the TRIPOD checklist among the three studies is shown in Supplementary Table S2.

#### 3.2.7 Risk of bias and applicability

**Figure 2:**
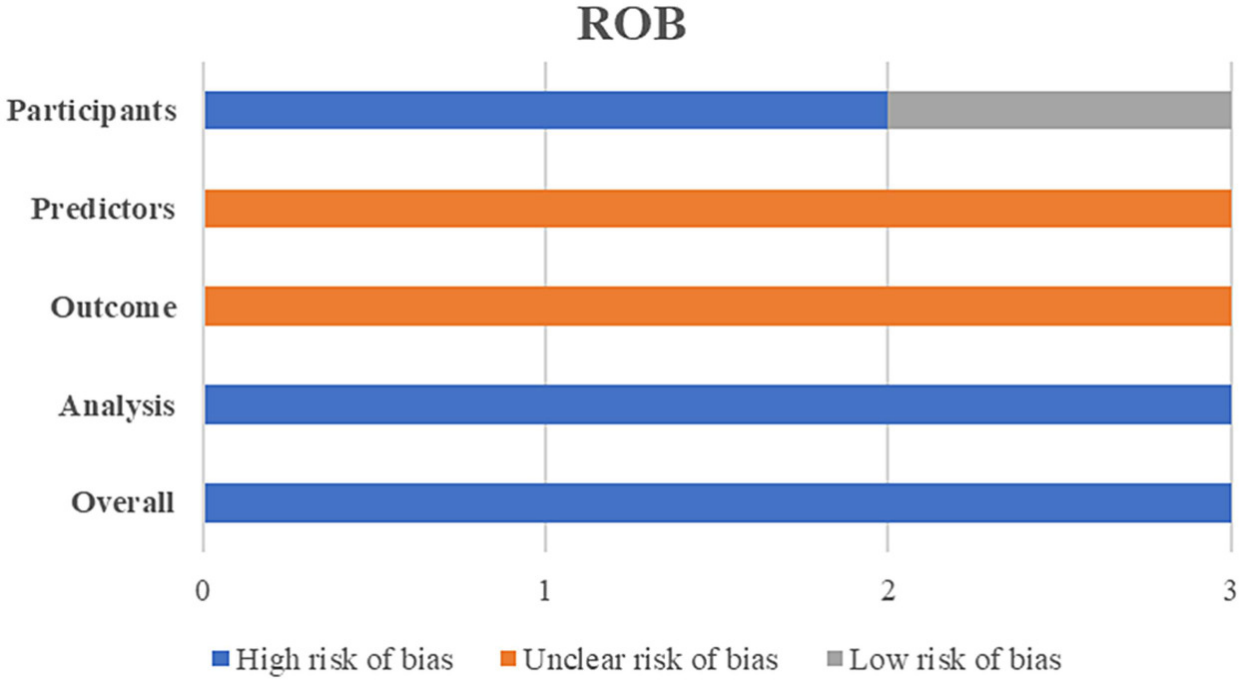
Risk of bias assessment for all included models (*N* = 3).

The PROBAST bias risk assessment results revealed that all three included models had a high risk of bias (Figure 2). Among the four assessed dimensions, the statistical analysis dimension had the worst assessment results, with all three models exhibiting a high risk of bias. Detailed scores for each dimension are shown in Supplementary Table S1.

**Figure 3:**
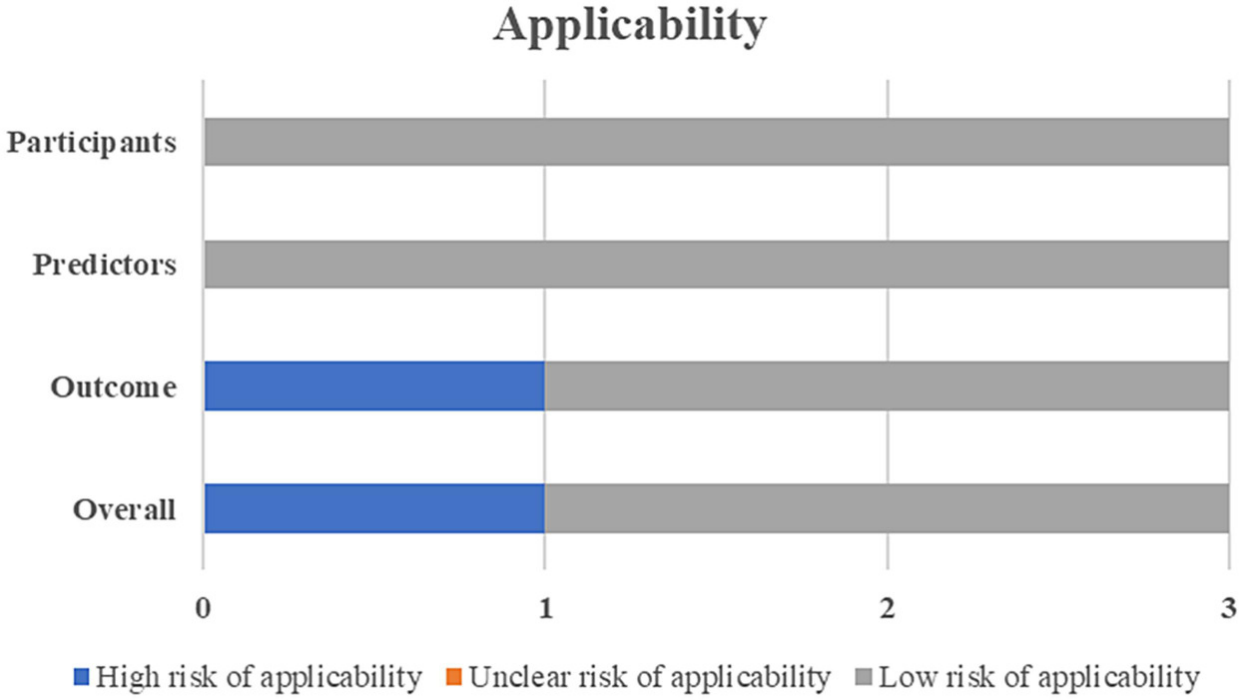
Applicability assessment for all included models (*N* = 3).

One study was deemed to have a high risk for applicability due to the lack of external validation (Figure 3).

## 4 Discussion

This review comprehensively assessed the current state of predictive models designed to forecast NIs in ICU child patients. We identified three models and summarized their performance and the predictors used. Additionally, we conducted an in-depth analysis and identified several methodological shortcomings in developing and validating these predictive models.

All studies employed Logistic regression, a commonly used method for constructing models with binary classification variables, which likely reflects practical and contextual factors inherent to pediatric ICU research. On the one hand, Logistic regression generates odds ratios that are clinically intuitive, allowing clinicians to directly link individual predictors (e.g., “antibiotic use >7 days”) to infection risk (28, 29). This interpretability is particularly valued in neonatal and pediatric settings, where clinical decision-making often requires clear mechanistic links to justify interventions. On the other hand, the included studies had relatively small samples (411–672 participants), with event rates as low as 5.1% (29). Logistic regression is robust to small datasets, remains a default due to its low computational demand, and familiarity among researchers. And avoids overfitting compared to complex models (e.g., machine learning), which require larger samples to generalize (28). However, the issue of multicollinearity was not addressed in any of the models, which may compromise model performance. Machine learning (ML) techniques, such as random forest (RF), artificial neural networks (ANN), LASSO, and eXtreme gradient boosting (XGBoost), have demonstrated robust capabilities in handling high-dimensional data and strong generalization abilities (32). Therefore, in developing models to predict the impact of NIs in ICU child patients, it is advisable to explore alternative algorithms like RF, LASSO and XGBoost. Pediatric ICU data, especially for critically ill children, often suffer from small sample sizes and limited event rates (e.g., 5.1% for umbilical vein catheter-related infections (29), which constrain traditional statistical methods like logistic regression. ML techniques offer unique advantages in this context: Handling small datasets with regularization: (1) Algorithms such as LASSO regression and elastic net incorporate built-in regularization, which penalizes overfitting by shrinking coefficients of less relevant predictors. This is critical for pediatric data, where limited events (e.g., 34 infections in 672 NICU patients (29) increase the risk of overfitting with conventional methods. (2) Leveraging feature interactions without large samples: ML models (e.g., random forests, gradient boosting) automatically capture non-linear relationships and interactions between predictors (e.g., the combined effect of low birth weight and prolonged antibiotic use (28, 29) without requiring the large sample sizes needed to validate such interactions in regression models. This is valuable given the complex, multifactorial nature of pediatric NI risk. If the performance of models developed using a single algorithm is unsatisfactory, ensemble learning methods could be employed to create ensemble models. These models automatically model non-linear relationships and interactions without manual transformation, capturing complex patterns in pediatric data, integrate the strengths of multiple algorithms, significantly enhancing prediction accuracy (33).

Most studies directly deleted missing values, leading to incomplete reporting. This approach can introduce bias, as missing data can distort model performance if correlated with other variables (34). Missing value imputation methods include deletion, simple imputation, multiple imputation, and algorithmic imputation. Multiple imputation (35) and Miss Forest (36) are currently more recommended. About half of the studies also transformed continuous variables into binary or multi-class classifications. While this simplifies clinical decision-making, it can result in loss of data information and reduced prediction performance (37). The choice of method should be guided by the study's purpose, the data characteristics, and the intended application.

Three studies selected predictors using univariate analysis. However, with the advancement of modeling methods, predictor selection is crucial for model quality and effectiveness. Predictors should be chosen by combining clinical literature with data-driven approaches. Relying solely on statistical significance for predictor selection can lead to overfitting and overly optimistic performance measures (38). Variable selection methods can be categorized into filter, wrapper, and embedding (39). Regularized regression (penalized models or shrinkage methods) can help reduce overfitting for data with many features or multicollinearity. Machine learning algorithms, such as random forest, can also be used for predictor selection and dimensionality reduction (40), thereby improving research quality. The predictors in the models identified in this review varied substantially based on the outcomes and study population, demonstrating the multifactorial nature of risk factors associated with NIs in ICU child patients. For frequently occurring predictors (included in 2 studies): Antibiotic use reflecting the well-established link between prolonged antibiotic exposure and increased risk of NIs, particularly MDRO infections (28, 30). Birth weight highlights the critical role of immature immune function and fragile skin barrier in neonatal susceptibility to infections (5, 6). Indwelling catheters were featured in both NICU and PICU models, consistent with the known association between invasive devices and infection risk due to disruption of physical barriers and pathogen colonization (4, 41). Furthermore, the sample size is intrinsically linked to the variables under consideration. Beyond the conventional 10-EPV (events per variable) estimation approach, advanced sample size calculation tools have been specifically developed to more accurately estimate the required sample size for clinical prediction models (42).

Two studies conducted internal and external validation, while another combined model construction with internal validation. Calibration, which assesses whether the predicted outcome matches the observed outcome, was investigated for all models. Poorly calibrated models can mislead decision-making processes (43). Predictive models often perform better on development data than external validation data, but external validation is more convincing than internal validation (44). Therefore, models should be validated using different datasets whenever possible to ensure generalizability. Validation studies should verify that the model's performance on new data is comparable to its performance on the development data (45), and model usefulness should be assessed through clinical judgment.

The included studies exhibited inadequate model presentation and incomplete regression equations. Poor presentation wastes research resources and hinders future activities such as validation, updating, and clinical application. Various forms can be used for model presentation, including scoring systems, nomograms, web calculators, and mobile apps. In this study, two models were presented using nomograms, while one model adopted a scoring table. None of the included studies reported converting their models into bedside scoring systems, online calculators, or EHR-embedded alert formats critical for real-time decision-making in busy ICUs. None of the studies reported formal usability testing with clinical end-users (e.g., ICU nurses, physicians). The limited attention to model presentation and usability in the included studies reflects a common focus on methodological rigor (e.g., discrimination, calibration) over practical implementation in early-stage model development. The Transparent Reporting of a Multivariable Prediction Model for Individual Prognosis or Diagnosis (TRIPOD) guidelines, published in 2015 in multiple well-known journals (19, 31), aim to standardize the reporting of prediction model research to ensure transparency and reproducibility. Although all three included studies were published after 2015, none explicitly stated adherence to the TRIPOD statement. Adhering to TRIPOD guidelines can significantly enhance transparency and reproducibility. We strongly recommend that researchers submit the TRIPOD checklist when submitting manuscripts to facilitate evaluation by journal editors and reviewers. Full compliance with these standards will ensure scientific rigor in model development and improve the model's applicability in clinical settings.

Most studies developed new models with excellent predictive power. However, these models consistently exhibited a high risk of bias (ROB). Among the two studies (28, 29) based on a retrospective design, there was a high risk of bias in the assessment of study subjects. None of the three studies reported whether the predictors were assessed without knowledge of the outcome data or whether all predictors were available at the time the model was intended to be used, or whether the outcome was determined without knowledge of the predictor information. Thus, the risk of predictor and outcome bias was rated as “unclear.” All models had issues in statistical analysis, such as insufficient sample size, inadequate consideration of overfitting, handling of missing data, and unclear treatment of continuous variables. This can result in good performance on the training set but poor performance on the test set or in real-world applications (44). Overfitting means that while the model learns the training data well, it cannot generalize to new data effectively, significantly reducing its usefulness and reliability (46). Therefore, identifying child patients who could benefit from interventions to prevent NIs remains a critical public health strategy. Predictive models used with clinical decision support have been shown to improve patient outcomes (47) and should be considered when deploying risk models in ICU child patients.

Last but not least, it is essential to evaluate models' impact in practice. In our review, two studies were conducted in NICUs and one in a PICU, with populations from China and India only. The baseline infection rates, pathogen spectra, and resource availability in the study settings (China and India) exhibit distinct characteristics that may constrain the external validity of the models. The included studies were conducted in high-volume tertiary centers, where the burden of NIs may differ from other settings. For example, the NICU studies from China reported 16.2% (89/549) and 5.1% (34/672) infection rates for MDRO infections and umbilical vein catheter-related bloodstream infections, respectively (28, 29), while the Indian PICU study reported a HAI rate of 23.1% (95/411) (30). The models focus on region-specific pathogens: Zhou et al. (28) targeted MDROs, which are prevalent in Asian tertiary centers with high antibiotic usage; Miao et al. (29) focused on umbilical vein catheter-related pathogens, relevant to neonates in resource-intensive NICUs; and Saptharishi et al. (30) used HAI definitions aligned with CDC criteria but in an Indian PICU, where pathogen profiles may include more community-acquired or drug-resistant strains due to limited antimicrobial stewardship (11). The included models incorporate predictors tied to local resource access, such as “antibiotic use >7 days” (28), “mechanical ventilation history” (29), and “indwelling catheter presence” (30). Conversely, resource-rich HICs may prioritize different predictors (e.g., immunocompromised status, advanced organ support) not captured in the current models, further limiting cross-context applicability. Based on the available evidence, none of the identified models are currently validated for global use. Their applicability may be restricted to settings with similarities to the original study populations.

We additionally searched for studies that implemented NI prediction models in child patients in ICUs, but we could not identify any references. However, evaluating whether introducing a prediction model changes to care, e.g., increases interventions and improves outcomes, e.g., reduces the incidence of NIs, will be necessary for future investigations. Based on these methodological shortcomings, we make the following recommendations. First, models should be externally validated several times in different populations, and sample sizes must be adequately considered. Second, when data are missing, interpolation should be performed via multiple interpolations or machine learning. Third, predictive variables with incremental solid values should be mined based on clinical feasibility and applicability, and preventing overfitting should be emphasized in the predictive model. At the same time, we also need to recognize the importance of diversified evaluation, as far as possible, sensitivity, specificity, the calibration index, the net benefit, and DCA for comprehensive evaluation. Finally, model development is strongly recommended to adhere to the TRIPOD process. Only complete, full, and transparent reporting of all aspects of a prediction model can its risk of bias and potential usefulness be adequately assessed.

The distinction between NICU and PICU populations introduces critical variability that limits cross-setting generalizability: Developmental stage: NICU populations consist of neonates, often premature, with immature immune systems, fragile skin barriers, and prolonged exposure to invasive interventions (e.g., umbilical catheters) (5, 6). In contrast, PICU patients are older children (1 month to 12 years (30) with diverse comorbidities and varying degrees of immune competence. Risk factors: NICU models emphasize neonatal-specific predictors such as birth weight <1,500 g (29) and maternal age (28), which are irrelevant to older PICU patients. PICU models, meanwhile, focus on factors like intubation need and PRISM III scores (30), reflecting the acuity of critical illness in older children. Infection dynamics: Neonates in NICUs are disproportionately susceptible to device-related infections (e.g., umbilical vein catheter sepsis (29), whereas PICU patients face broader HAI risks linked to mechanical ventilation and immunomodulatory therapies (30). These differences mean that models developed for one setting (e.g., NICU) may not accurately identify high-risk patients in the other (e.g., PICU), as the underlying pathophysiology and risk profiles diverge.

Outcome variability further constrains generalizability: MDROs as a subset of HAIs: MDRO infections (28) represent a specific, drug-resistant subgroup of HAIs, driven by factors like prolonged antibiotic exposure (28, 30). This focus limits applicability to settings with high antibiotic stewardship or low MDRO prevalence. Device-specific vs. broad HAIs: The umbilical vein catheter-associated infections modeled by Miao et al. (29) are niche outcomes relevant only to neonates with invasive devices, whereas Saptharishi et al.'s (30) broader HAI definition (encompassing all healthcare-acquired infections) has a wider scope but may mask device-specific risks. Diagnostic criteria: Variations in diagnostic standards (e.g., CDC criteria for HAIs (30) vs. Chinese Ministry of Health criteria for MDROs (28) complicate cross-study comparisons and reduce the transferability of findings to settings with different surveillance protocols. Thus, models targeting MDROs or device-specific infections cannot be generalized to predict all HAIs, and vice versa, as their underlying etiologies and risk factors differ.

This review aimed to identify and evaluate predictive models for NIs in ICU child patients to inform clinical decision-making. However, we cannot recommend any specific model for several reasons. First, nearly all reviewed models exhibited a high risk of bias, and the included models require further external validation. Additionally, significant heterogeneity among the models, non-standardized statistical analysis methods, and incomplete data in model reports all contribute to the challenge of selecting the optimal model. While no existing model meets the criteria for routine clinical application, we propose the following interim strategies to address the urgent need for NI risk stratification in critically ill children: (1) Adapting validated adult ICU models with pediatric-specific adjustments: (A) Adult NI prediction models (e.g., those incorporating variables like invasive device duration, antibiotic exposure, and comorbidity burden) (4, 41) could serve as a foundation, with key modifications to account for pediatric physiology. (B) Adjusting for developmental factors [e.g., replacing adult age with postmenstrual age in neonates (5, 6)]. Incorporating pediatric-specific variables identified in our review, such as birth weight (28, 29), umbilical catheter use (29), and PRISM III scores (30). (C) Calibrating risk thresholds to reflect higher baseline vulnerability in children [e.g., lower thresholds for initiating interventions in NICUs due to immature immune function (7)]. (2) Implementing structured clinical risk assessment tools: Given the paucity of validated models, we recommend using consensus-based checklists that integrate the most consistent predictors from included studies: Antibiotic use exceeding 7 days (28, 30), presence of indwelling catheters (29, 30), and (in neonates) low birth weight (28, 29).

To address the “methodological robustness” gap highlighted in our conclusions, we specify the following priorities for future research, based on PROBAST and TRIPOD guidelines (19, 27). (1) Rigorous model development: (A) Sample size and event rates: Adhere to modern standards [e.g., minimum 100–200 events (42)] to avoid overfitting, particularly for rare outcomes like MDRO infections (28). (B) Handling missing data: Use advanced imputation methods [e.g., multiple imputation (35) or MissForest (36)] instead of complete-case analysis, which risks bias (28, 29). (C) Predictor selection: Combine clinical expertise (e.g., neonatology input for NICU models) with data-driven methods [e.g., LASSO regression, random forest variable importance (39, 40)] to avoid over-reliance on univariate analysis (28–30). (2) Comprehensive validation: (A) External validation across diverse settings: Validate models in geographically distinct centers (e.g., comparing tertiary vs. community hospitals) and populations (e.g., NICUs vs. PICUs) to assess generalizability (34, 44). (B) Temporal validation: Include longitudinal cohorts to ensure stability of model performance over time, as demonstrated in two included studies (28, 29), but expanded to multi-year follow-up. (3) Advanced modeling techniques: Explore machine learning approaches [e.g., random forest, XGBoost (32, 33)] to capture non-linear relationships between predictors [e.g., interaction between antibiotic use and indwelling catheters (28, 30)] that traditional logistic regression may miss. Use ensemble methods to integrate the strengths of multiple models, potentially improving performance beyond single-algorithm approaches (33). (4) Enhanced transparency and clinical utility: (A) Strictly adhere to TRIPOD guidelines (19, 31), including full reporting of regression equations, calibration metrics, and validation protocols (absent in all included studies (28–30). (B) Develop user-friendly tools (e.g., electronic health record-integrated calculators, mobile apps) and conduct usability testing with ICU nurses/physicians to ensure real-world applicability (47).

### Strengths and limitations

This study is the first to conduct a comprehensive and integrated assessment of predictive models for NIs in ICU child patients. We provided valuable information for primary healthcare systems and clinical healthcare professionals through an extensive literature search, meticulous screening, and standardized data extraction. This approach lays the foundation for more effective construction and external validation of future predictive models. Furthermore, this study conducted a risk of bias and applicability assessment of the prediction models using the PROBAST tool, another significant strength.

This review is subject to certain limitations. Firstly, the literature search was confined to computerized databases and restricted to materials published in English and Chinese, potentially excluding relevant studies in other languages. Secondly, we could not perform meta-analyses or subgroup analyses due to the limited number of included studies and considerable heterogeneity in participant demographics, research settings, and outcomes. These variabilities could have affected the comparability and generalizability of the findings.

## Conclusions

We identified three predictive models (one study was conducted in the PICU). In contrast, two studies were carried out in NICUs, and most of the researchers reported excellent discrimination and calibration in their research. However, for various reasons, the risk of bias in nearly all the models was high. Consequently, this finding implies that the predictive performance of these models might be overestimated, their accuracy in practical application to the target population remains questionable, and currently, we cannot endorse any of these predictive models for clinical practice. High-performance predictive models can assist clinical nursing staff in the early identification of high-risk populations for NIs, enabling proactive interventions to reduce infection rates. With the development of modeling methods, the selection of predictors largely determines the quality and effectiveness of the model. Predictors should be selected by combining clinical literature reports with data-driven approaches. Future research on predictive models for NI risk in ICU child patients should adhere to methodological guidelines, prioritize practicality and cost-effectiveness in model evaluation, conduct large-scale external validation, and ultimately facilitate effective identification of NIs in critically ill children.
