# Supplementary material for: Predicting nosocomial infections in critically Ill children: a comprehensive systematic review of risk assessment models
Source: Front Pediatr. 2025 Sep 10;13:1636580. doi: 10.3389/fped.2025.1636580 (PMC12459274; doi:10.3389/fped.2025.1636580)
Supplement: Supplementary file 1 [file Supplementaryfile1.docx]

**Appendix 1**

According to the surveillance definitions of the Centers for Disease Control and Prevention (CDC), healthcare–associated infections (HAIs) are defined as local or systemic conditions resulting from an adverse reaction to the presence of an infectious agent(s) or its toxin(s), and there must be no evidence that the infection was present or incubating at the time of admission to the acute care setting. HAIs can be caused by endogenous or exogenous infectious agents. Endogenous sources are body sites such as the skin, nose, mouth, gastrointestinal tract, or vagina, which are usually habitats for microorganisms. Exogenous sources come from outside the patient, such as healthcare workers, visitors, medical equipment, or the healthcare environment.
