# Supplementary material for: Predicting nosocomial infections in critically Ill children: a comprehensive systematic review of risk assessment models
Source: Front Pediatr. 2025 Sep 10;13:1636580. doi: 10.3389/fped.2025.1636580 (PMC12459274; doi:10.3389/fped.2025.1636580)
Supplement: Supplementary file 2 [file Datasheet1.pdf]

Supplementary Table 1 Search strategy

**Pubmed**	**1.** ((((Intensive Care Units[MeSH Terms]) OR (Intensive Care Units[Title/Abstract])) OR (Intensive Care Unit[Title/Abstract])) OR (Unit, Intensive Care[Title/Abstract])) OR (ICU [Title/Abstract])<br>**2.** (((Infection Control[MeSH Terms]) OR (Control, Infection[Title/Abstract])) OR (transmiss*[Title/Abstract])) OR (nosocomial*[Title/Abstract])<br>**3.** ("model"[Title/Abstract] OR "prediction model" [Title/Abstract] OR "prediction rule" [Title/Abstract] OR "risk assessment model" [Title/Abstract] OR "risk prediction model" [Title/Abstract] OR "risk score"[Title/Abstract] OR "risk stratification" [Title/Abstract] OR "predictive model"[Title/Abstract] OR "predictive score"[Title/Abstract])<br>**4.** 1 AND 2 AND 3
**Embase**	#1 (model or prediction model or prediction rule or risk assessment model or risk prediction model or risk score or risk stratification or predictive model or predictive score).ab,at.<br>#2 (Intensive Care Units or Intensive Care Unit or Unit, Intensive Care or ICU).ab,at.<br>#3 (Infection Control or Control, Infection or transmiss* or nosocomial*).ab,at.<br>#4 #1 AND #2 AND #3
**Web of Science**	#1 TI=(model) OR TI= (prediction model) OR TI=(prediction rule) OR TI=(risk assessment model)OR TI=(risk prediction model) OR TI=(risk score) OR TI=(risk stratification) OR TI=(predictive model) OR TI=(predictive score)<br>#2 TI= (Intensive Care Units) OR TI=(Intensive Care Unit) OR TI=(Unit, Intensive Care) OR TI=(ICU)<br>#3 TI=(Infection Control) OR TI=(Control, Infection) OR TI=(transmiss*) OR TI=(nosocomial*)<br>#4 #1 AND #2 AND #3
**CNKI**	((SU=Intensive Care Units OR SU=ICU) AND (SU=Infection Control OR SU=nosocomial infection) AND (SU=model OR SU=prediction model OR SU=prediction rule OR SU=risk assessment model OR SU=risk score OR SU=risk stratification OR SU=predictive model OR SU=predictive score))
**Wanfang**	(Intensive Care Units or ICU) and (Infection Control or nosocomial infection) and (model or prediction model or prediction rule or risk assessment model or risk score or risk stratification or predictive model or predictive score)
**VIP**	(U= Intensive Care Units OR U=ICU) AND (U=Infection Control OR U=nosocomial infection) AND (U=model OR U=prediction model OR U=prediction rule OR U=risk assessment model OR U=risk score OR U= risk stratification OR U=predictive model OR U=predictive score)
