# Supplementary material for: Predicting nosocomial infections in critically Ill children: a comprehensive systematic review of risk assessment models
Source: Front Pediatr. 2025 Sep 10;13:1636580. doi: 10.3389/fped.2025.1636580 (PMC12459274; doi:10.3389/fped.2025.1636580)
Supplement: Supplementary file 3 [file Datasheet2.pdf]

Supplementary Table 1 Assessment of risk of bias

**Items**	**Zhou/2023**	**Miao/2023**	**L.G. Saptharishi/2016**
**1.Participants**			
1.1. Were appropriate data sources used, e.g., cohort, RCT, or nested case-control study data?	no / probably no	no / probably no	yes/ probably yes
1.2. Were all inclusions and exclusions of participants appropriate?	yes/ probably yes	yes/ probably yes	yes/ probably yes
**2.Predictors**			
2.1. Were predictors defined and assessed in a similar way for all participants?	yes/ probably yes	yes/ probably yes	yes/ probably yes
2.2. Were predictor assessments made without knowledge of outcome data?	no information	no information	no information
2.3. Were all predictors available at the time the model is intended to be used?	no information	no information	no information
**3.Outcome**			
3.1. Was the outcome determined appropriately?	yes/ probably yes	yes/ probably yes	yes/ probably yes
3.2. Was a prespecified or standard outcome definition used?	yes/ probably yes	yes/ probably yes	yes/ probably yes
3.3. Were predictors excluded from the outcome definition?	yes/ probably yes	yes/ probably yes	yes/ probably yes
3.4. Was the outcome defined and determined in a similar way for all participants?	yes/ probably yes	yes/ probably yes	yes/ probably yes
3.5. Was the outcome determined without knowledge of predictor information?	no information	no information	no information
3.6. Was the time interval between predictor assessment and outcome determination appropriate?	yes/ probably yes	yes/ probably yes	yes/ probably yes
**4.Analysis**			
4.1. Were there a reasonable number of participants with the outcome?	yes/ probably yes	no / probably no	yes/ probably yes
4.2. Were continuous and categorical predictors handled appropriately?	no / probably no	no / probably no	no / probably no
4.3. Were all enrolled participants included in the analysis?	yes/ probably yes	no / probably no	yes/ probably yes
4.4. Were participants with missing data handled appropriately?	no / probably no	no information	no / probably no
4.5. Was selection of predictors based on univariable analysis avoided?	no / probably no	no information	no / probably no
4.6. Were complexities in the data (e.g., censoring, competing risks, sampling of control participants) accounted for appropriately?	no information	no information	no information
4.7. Were relevant model performance measures evaluated appropriately?	yes/ probably yes	no / probably no	yes/ probably yes
4.8. Were model overfitting, underfitting, and optimism in model-performance accounted for?**	yes/ probably yes	no / probably no	yes/ probably yes

4.9. Did predictors and their assigned weights in the final model correspond to the results from the reported multivariable analysis?**	yes/ probably yes	no / probably no	yes/ probably yes

RCT = randomized controlled trial

*For further details, please see the explanation and elaboration document, available at Annals.org. and www.probast.org. Signalling questions are answered as yes, probably yes, probably no, no, or no information. ROB and concerns for applicability are rated as low, high, or unclear.

**Development studies only.

Supplementary Table 2 Completion status of the TRIPOD checklist among three studies

Section/Topic	Item		Checklist Item	Reporting Status		
**Title and abstract**				**Zhou/2023**	**Miao/2023**	**L.G. Saptharishi/2016**
Title	1	D;V	Identify the study as developing and/or validating a multivariable prediction model, the target population, and the outcome to be predicted.	√	√	√
Abstract	2	D;V	Provide a summary of objectives, study design, setting, participants, sample size, predictors, outcome, statistical analysis, results, and conclusions.	√	√	√
**Introduction**						
Background and objectives	3a	D;V	Explain the medical context (including whether diagnostic or prognostic) and rationale for developing or validating the multivariable prediction model, including references to existing models.	√	√	√
	3b	D;V	Specify the objectives, including whether the study describes the development or validation of the model or both.	√	√	√
**Methods**						
Source of data	4a	D;V	Describe the study design or source of data (e.g., randomized trial, cohort, or registry data), separately for the development and validation data sets, if applicable.	√	√	√
	4b	D;V	Specify the key study dates, including start of accrual; end of accrual; and, if applicable, end of follow-up.	√	√	√
Participants	5a	D;V	Specify key elements of the study setting (e.g., primary care, secondary care, general population) including number and location of centres.	√	√	√
	5b	D;V	Describe eligibility criteria for participants.	√	√	√

	5c	D;V	Give details of treatments received, if relevant.	Not appliable	Not appliable	Not appliable
Outcome	6a	D;V	Clearly define the outcome that is predicted by the prediction model, including how and when assessed.	√	√	√
	6b	D;V	Report any actions to blind assessment of the outcome to be predicted.	×	×	×
Predictors	7a	D;V	Clearly define all predictors used in developing or validating the multivariable prediction model, including how and when they were measured.	√	√	√
	7b	D;V	Report any actions to blind assessment of predictors for the outcome and other predictors.	×	×	×
Sample size	8	D;V	Explain how the study size was arrived at.	√	√	√
Missing data	9	D;V	Describe how missing data were handled (e.g., complete-case analysis, single imputation, multiple imputation) with details of any imputation method.	×	×	×
Statistical analysis methods	10a	D	Describe how predictors were handled in the analyses.	√	√	√
	10b	D	Specify type of model, all model-building procedures (including any predictor selection), and method for internal validation.	√	√	√
	10c	V	For validation, describe how the predictions were calculated.	√	√	×
	10d	D;V	Specify all measures used to assess model performance and, if relevant, to compare multiple models.	√	√	√
	10e	V	Describe any model updating (e.g., recalibration) arising from the validation, if done.	√	√	√
Risk groups	11	D;V	Provide details on how risk groups were created, if done.	Not appliable	Not appliable	Not appliable
Development vs. validation	12	V	For validation, identify any differences from the development data in setting, eligibility criteria, outcome, and predictors.	√	√	×
**Results**						
Participants	13a	D;V	Describe the flow of participants through the study, including the number of participants with and without the outcome and, if applicable, a summary of the follow-up time. A diagram may be helpful.	√	√	√
	13b	D;V	Describe the characteristics of the participants (basic demographics, clinical features, available predictors), including the number of participants with missing data for predictors and outcome.	√	√	√

	13c	V	For validation, show a comparison with the development data of the distribution of important variables (demographics, predictors and outcome).	√	√	×
Model development	14a	D	Specify the number of participants and outcome events in each analysis.	√	√	√
	14b	D	If done, report the unadjusted association between each candidate predictor and outcome.	√	√	√
Model specification	15a	D	Present the full prediction model to allow predictions for individuals (i.e., all regression coefficients, and model intercept or baseline survival at a given time point).	√	√	√
	15b	D	Explain how to the use the prediction model.	√	√	√
Model performance	16	D;V	Report performance measures (with CIs) for the prediction model.	√	√	√
Model-updating	17	V	If done, report the results from any model updating (i.e., model specification, model performance).	Not appliable	Not appliable	Not appliable
**Discussion**						
Limitations	18	D;V	Discuss any limitations of the study (such as nonrepresentative sample, few events per predictor, missing data).	√	√	√
Interpretation	19a	V	For validation, discuss the results with reference to performance in the development data, and any other validation data.	√	√	×
	19b	D;V	Give an overall interpretation of the results, considering objectives, limitations, results from similar studies, and other relevant evidence.	√	√	√
Implications	20	D;V	Discuss the potential clinical use of the model and implications for future research.	√	√	√
**Other information**						
Supplementary information	21	D;V	Provide information about the availability of supplementary resources, such as study protocol, Web calculator, and data sets.	√	×	×
Funding	22	D;V	Give the source of funding and the role of the funders for the present study.	Not appliable	Not appliable	Not appliable

*Items relevant only to the development of a prediction model are denoted by D, items relating solely to a validation of a prediction model are denoted by V, and items relating to both are denoted D;V. We recommend using the TRIPOD Checklist in conjunction with the TRIPOD Explanation and Elaboration document.
